# Percutaneous Endoscopic Gastrostomy Tubes Can Be Considered Safe in Children: A Single-Center 11-Year Retrospective Analysis

**DOI:** 10.3390/medicina57111236

**Published:** 2021-11-12

**Authors:** Antonia Jeličić Kadić, Tea Radošević, Vanda Žitko, Ranka Despot, Zenon Pogorelić, Carlos Martin Llorente Muñoz, Edita Runjić, Tanja Kovačević, Tatjana Ćatipović Ardalić, Branka Polić, Joško Markić

**Affiliations:** 1 Department of Pediatrics, University Hospital of Split, 21 000 Split, Croatia; jelicic.antonia@gmail.com (A.J.K.); vzitko@kbsplit.hr (V.Ž.); ranka.despot11@gmail.com (R.D.); editarunjic@gmail.com (E.R.); tanjakovacevic74@yahoo.com (T.K.); tatjana.ardalic13@gmail.com (T.Ć.A.); branka.polic1@gmail.com (B.P.); 2 School of Medicine, University of Split, 21 000 Split, Croatia; tea2radosevic@gmail.com (T.R.); zpogorelic@gmail.com (Z.P.); 3 Department of Pediatric Surgery, University Hospital of Split, 21 000 Split, Croatia; 4 Surgical Clinic Medix-Muñoz, 28 000 Madrid, Spain; llorentecm@gmail.com

**Keywords:** percutaneous gastrostomy, children, malnutrition, treatment outcome, complications

## Abstract

*Background and Objectives:* When the human body is disabled to naturally ingest food through the mouth, enteral or parenteral nutritional support should be started. Percutaneous gastrostomy (PEG) is a flexible feeding tube that is inserted into the stomach through the abdominal wall in patients who will need long-term enteral nutrient intake. The aim of this study is to analyze clinical characteristic of children at the time of PEG placement as well as to determine indications, complications and outcomes associated with PEG at the Department of Pediatrics of the University Hospital of Split. *Materials and Methods*: Retrospective analysis of the medical records of patients treated from 2010 to 2020 was performed. The following data were collected from medical records: age, gender, information about nasogastric feeding before PEG placement, indication for PEG insertion, duration of PEG, procedure-related complications and treatment outcomes. Malnutrition was determined according to the z-score range for BMI for age and sex. According to the indication for PEG placement, patients were divided into five categories: central nervous system (CNS) diseases, neuromuscular diseases, genetic disorders, metabolic diseases, and group of children with polytrauma. *Results*: A total of 40 patients with median age of 110 months were included in study. At the time of PEG placement, most patients had deviations in body weight and height compared to expected values for age and sex. The most common underlying diagnoses were diseases of the central nervous system. Minor complications were found in 13 (35%) of patients. One patient (2.7%) developed major complication (gastrocolic fistula) and consequently underwent reoperation. The median duration of PEG in patients with complications before the need for replacement was 27 months, and in patients without complications, 43 months. *Conclusions*: Negative deviations of z-score body weight, body height, and body mass index could indicate the need for possible earlier placement of PEG. PEG can be considered as a safe therapeutic option in children since PEG-related complications, mostly in minor forms, were found in a small number of patients.

## 1. Introduction

At the moment when the human body is disabled to naturally ingest food through the mouth, enteral or parenteral nutritional support is approached. Enteral feeding is a term that refers to the direct introduction of liquid or mushy nutritional support into the stomach or small intestine while avoiding the mouth and esophagus. Enteral food intake can be performed via tubes that are placed through the mouth or nose into the stomach or small intestine, and are called orogastric, nasogastric or nasoenteral tubes. The second form of enteral intake is by using gastrostomies or jejunostomies, which are tubes placed through the skin directly into the stomach or small intestine [1]. Whenever possible, enteral over parenteral intake is preferred due to possible consequences of placing through an intravenous line, significant costs of parenteral nutrition, and lack of intestinal stimulation and consequently, the disruption of the intestinal barrier in parenteral feeding [2]. Timely initiation of enteral nutrition is optimal for avoiding complications of malnutrition [3]. Percutaneous gastrostomy (PEG) is the name of a flexible feeding tube that is inserted into the stomach through the abdominal wall [4]. It is the best way of feeding for patients who will need long-term enteral nutrient intake and who have a functional gastrointestinal system at the same time [5].

Although originally described in the pediatric population, it was soon introduced as a feeding aid in adult patients as well [6]. The most common indications for the placement of PEG are dysphagia and feeding difficulties that arise because of various diseases of the central nervous system [7,8]. Other conditions that lead to dysphagia and slow progression of children and consequently stand out as indications for the placement of percutaneous gastrostomies are congenital anomalies of the oropharynx, unclassified neurological diseases, chromosomal and metabolic diseases [7,8]. Additionally, PEG is an efficient way of feeding patients who need long-term enteral nutrition at home because it carries considerably lower risk of developing complications than feeding through a nasogastric tube. PEG feeding can be used when oral food intake, despite undisturbed swallowing, does not provide sufficient energy support to the body. PEG can also be placed in patients with maxillary injury, patients with mental disabilities, and patients who are in a permanent vegetative state or near the end of life [9]. On the other hand, contraindications for PEG placement are: serious coagulation disorders, hemodynamic instability, sepsis, severe ascites, peritonitis, abdominal wall infection at the placement site, peritoneal carcinomatosis, lack of a safe tract for percutaneous insertion, gastric outlet obstruction, severe gastroparesis, history of total gastrectomy, prolonged ventilation assistance and lack of informed consent [5].

The gastrostomies can be placed using laparoscopic or percutaneous approaches and it is not yet clear which of the used techniques is most effective and safest in children [10]. There are three different techniques for PEG placement, the per oral pull technique, the per oral push technique and direct percutaneous technique [11]. There are no significant differences in complications and efficiency between the ‘pull’ and ‘push’ techniques [12]. Since all these methods use general anesthesia that carries its risks, there is a need for a method that would avoid this. In the last several years, a one-step gastrostomy technique has been increasingly used.

It is safe to start feeding four hours after PEG placement [11,13]. The gastrostomy tube has to be rinsed before and after each meal and medication must be administered to prevent clogging and bacterial growth [5]. In addition, it is necessary to push the gastrostomy tube 2–4 cm to the stomach daily and rotate it between the fingers to prevent it from ingrowing into the submucosa [3]. It is not clear what type of the enteral feed used after the procedure is best or what kind of routine use of a clear fluid test or dilute or hypotonic feed after the procedure is most suitable.

Although the PEG is considered to be very safe way of enteral nutrition, there is a possibility of developing complications. Risks factors for development remain unknown. Patient mortality rates may increase in the presence of PEG-related complications [14]. Minor complications include erythema, local infection, formation of granulation tissue, external leakage, occlusion, dislocation, transient gastroparesis and ulceration of the gastric mucosa [3,5,7]. Major complications are very rare and can occur in these forms: cellulitis, peritonitis, necrotizing fasciitis, ileus, pneumoperitoneum, perforation of the stomach or esophagus, gastrocolic fistulas, granulation, permanent external and internal leakage, bleeding, subcutaneous abscess, aspiration pneumonia, systemic infections, and sepsis [3,5,7]. The most common complications are well described but there is a lack of clear methods on how these complications could be avoided.

The aim of this study is to analyze clinical characteristics of children at the time of PEG placement as well as to determine indications, complications and outcomes associated with PEG.

## 2. Materials and Methods

### 2.1. Patients

The study was conducted at the University Hospital of Split, Department of Pediatrics in Split, Croatia. This was a retrospective, single-center study; data were collected from archived hospital patient records. Patients (age < 18 years) who underwent PEG placement at the Department of Pediatrics between January 2010 and December 2020 were included. Patients with missing data were excluded from study. The following data were collected from patient medical records: age, gender, information about nasogastric feeding before PEG placement, indication for PEG insertion, duration of PEG, procedure-related complications and treatment outcomes (January 2021). Body mass index (BMI) was calculated from the measured body height (BH) and body mass (BM) using the following equation: BMI = BM (kg)/BH^2^ (m). Body mass, height and BMI were standardized using a CDC calculator and expressed as z-value. According to the indication for PEG placement, patients were divided into five categories: central nervous system (CNS) diseases, neuromuscular disease, genetic disorders, metabolic diseases, and group with children with polytrauma. The group of patients with CNS diseases includes patients with cerebral palsy, lissencephaly, neuronal ceroid lipofuscinosis, hypoxic–ischemic encephalopathy and epilepsy. Malnutrition was determinate according to the z-score range for BMI for age and sex. According to World Health organization standards and references for BMI, patients were divided into four groups: normal weight (z-score from −2 to 1), overweight (z-score ≥ 1 to 2), underweight (z-score −3 ≤ −2) and severe underweight (z-score ≤ −3) [15]. The procedure of PEG placement was performed using the pull method. This method includes two physicians, a pediatric gastroenterologist (V.Z.) for endoscopic guidance and the pediatric surgeon (Z.P.) for percutaneous interventions.

### 2.2. Outcomes of the Study

Primary outcome was an indication for PEG insertion. Duration of nasogastric feeding before PEG placement, duration of PEG, procedure-related complications and treatment outcomes were selected as secondary outcomes.

### 2.3. Description of Procedure

The procedure was performed using the “pull” technique. This technique requires two physicians: a gastroenterologist for endoscopic guidance (V.Z.) and a surgeon for percutaneous interventions (Z.P.). All patients were under general anesthesia. After induction of anesthesia and tracheal intubation by endotracheal tube (Curity™ Oral/Nasal Tracheal Tube Cuffed, COVIDien, Mansfield, MA, USA) standard intraoperative monitoring including arterial blood pressure, electrocardiograph, heart rate, and peripheral oxygen saturation (Draeger-Perseus A500 Anesthesia Device Monitor, Denver, CO, USA) were conducted. To measure the depth of anesthesia, a bispectral index monitoring system (BIS™ brain monitoring System, COVIDien, San Jose, CA, USA) was used. The patient was placed in a supine position on the operating table. For visualization of the best place for the PEG tube, the standard esophagogastroduodenoscopy was performed. After visualization of the stomach, the surgeon inserted a needle with a string which the gastroenterologist grasped using the scope and pulled out through the mouth. Subsequently the string was fixed to the external end of the feeding tube and the tube was pulled through the mouth to the esophagus, stomach, and then out through the abdominal wall. We used two PEG tubes; Freka PEG Set Gastric (Fresenius Kabi, Bad Homburg, Germany) and Flocare PEG Set (Nutricia Medical Devices, Schipol, The Nederlands).

### 2.4. Follow-Up

All the procedures were performed during a hospital stay. Close follow-up was undertaken for at least 7 days after PEG placement, then once a month for the first 3 months, and once every three months during the first year. After that, the follow-up was as needed, often to optimize a diet according to their nutritional status. Parents or caretakers were educated in managing feeding tubes and enteral feeding pumps at their homes. They were capable of taking aftercare of the feeding tube: flushing the feeding channel, feeding and giving medications separately, cleaning the puncture site (stoma) and tube as well as rotating it in the stoma. Additionally, they were advised to report any changes regarding stoma such as redness, soiling, bleeding, forming granulomas, as well as obstructions of tubes or displacements. In any of these circumstances they were instructed to contact our department regardless of the working hours.

### 2.5. Statistical Analysis

Microsoft Excel formulas were used for descriptive statistics (Microsoft Inc., Redmond, WA, USA). Statistical analyses were conducted using MedCalc software (MedCalc software, Mariakerk, Belgium; version 11.5.1.0). Comparative analyses were performed with Mann–Whitney U test and *p* values < 0.05 were considered significant.

## 3. Results

Over an eleven-year period, the PEG tube was placed in 42 children. Two children were excluded from analysis due to missing data. The youngest patient was 9 months old and the oldest was 210 months old (*n* = 40, median age was 110 months, (IQR 53, 158). There were 16 (40%) females and 24 (60%) males. Median BM before PEG placement in females was 22 kg (IQR 15, 29) and for males 19 kg (IQR 11, 34). Since there was no statistically significant difference between males and females in age, BM, BH and BMI at the time of PEG placement further analysis was conducted for all patients together (Table 1). The z-value of BMI was analyzed in 33 patients. According to the z-value of BMI, 39.4% (*n* = 13) patients had a normal body mass, 18.2% patients were overweight (*n* = 6), 15.2% (*n* = 5) patients were underweight and 27.2% (*n* = 9) were severely underweight. The main indication for PEG placement in patients who were overweight was “de novo dysphagia”. Among these patients, two had CNS disease, two had polytrauma, one had metabolic disease and one had neuromuscular disease. More than half of these patient needed permanent mechanical ventilation due to respiratory insufficiency. Prior to the PEG placement, nasogastric feeding was used in 30 (75%) patients. The median time of nasogastric feeding before PEG placement was 11 months IQR 5.5, 31.75).

The most common indication for PEG placement was underlying CNS disease (*n* = 23, 57.5%), followed with neuromuscular diseases (*n* = 7, 17.5%), polytrauma (*n* = 4, 10%), genetic disorders (*n* = 3, 7.5%) and metabolic diseases (*n* = 3, 7.5%). More than half of the patients with CNS disease had cerebral palsy (*n* = 14), and median age of these patients at the time of PEG placement was 150 months (IQR 121.8, 179.3).

During the follow-up period, information about outcomes and complications were available for 37 patients, and in 27 of them (73%) there was no need for the PEG tube replacement, in 6 patients (16.2%) PEG was replaced with GastroTube, in 3 patients (8.1%) PEG was changed, and only in 1 patient (2.7%), PEG was removed. During the follow-up period, five (13.5%) patients died (none of them died from PEG complications). Most of the patients (*n* = 24, 65%) had no complications, while 13 patients (35%) developed one or more complications. A total of 18 different complications were reported, 17 minor complications (acute wound inflammation, mechanical difficulties in PEG function, granulation tissue/scarring formation) and one major complication (gastrocolic fistula) (Table 2). Four patients had more than one complication.

The most common complications were local infection and granulation. The median duration time of PEG in all patients was 33 months (IQR 19, 60). In the patients without complications, it was 43 months (IQR 18, 63), and in patients with complications it was 27 months (IQR 20, 53).

Surgical complications according to Clavien–Dindo classification are shown in Table 3 [16]. Nine patients (53%) developed complications in the early postoperative period while in eight (47%) of them, complications occurred in late postoperative period.

A child with gastrocolic fistula presented 12 months after initial surgery with mechanical difficulties related to PEG. The pediatrician–gastroenterologist tried to replace PEG with a gastric tube, but failed. Esophagogastroscopy and computed tomography confirmed the existence of gastrocolic fistula. Open surgery was indicated, fistula resection and terminoterminal anastomosis of the colon were performed. The patient recovered quickly without any further complications.

There is no statistically significant difference between age (z = 1.64; *p* = 0.101), BMI (z = 0.081; *p* = 0.935), BMI z-values (z = 0.345; *p* = 0.730), z-values for BM (z = 0.843; *p* = 0.399) and z-values for BH (z = 0.0; *p* = 0.999) between patients with and without PEG-related complications. Additionally, in a group of patients who had not PEG replacement there is no statistically significant difference between patients who had complications related to PEG and those who did not have complications related to PEG in any of the studied parameters (age, BMI, z-value for BMI, z-value for BM and z-value for BH) (*p* > 0.05) (Table 4). Patients who had complications and PEG tube replacement were younger (median age 55 months, IQR 39.75, 82.25) than patients who had PEG replacement and did not have complications (age 133 months).

## 4. Discussion

This study includes pediatric patients aged from 9 to 210 months (median 110 months). More than 40% of them, at the time of PEG insertion, had malnutrition (according to z-score of BMI), and the most common indication for PEG insertion was CNS disease. Our study also shows that patients who had PEG replacement and complication were younger than those without complications. Percutaneous gastrostomy is an alternative way of feeding when oral intake is not possible or when there are difficulties in swallowing. It is an adequate method of enteral nutrition for patients whose dietary intake has been inadequate for more than 4–6 weeks and who have a functional gastrointestinal tract at the same time [4].

The ESPGHAN guidelines [3] recommend that the decision for PEG insertion should be made by an individualized approach by a multidisciplinary team. Significant advantages of PEG placement in children with malnutrition or children with eating disorders are well described [3,17]. The advantages are not only in nutritional composition of food intake but also in body weight and laboratory parameters [18,19]. Suh et al. showed significant improvement in weight-for-height *z*-score and height-for-weight *z*-score in children with cerebral palsy 6 months after PEG placement [18]. Additionally, the significant improvement in calorie intake is also described after PEG tube placement [18]. The limitation of this study was a small number of enrolled children with cerebral palsy (16 children). Viktorsdottir et al. in their retrospective study also showed a significant improvement in weight gain 12 months after PEG placement [19]. Malnutrition is also an important risk factor for developing nosocomial infections. Malnourished patients acquired nosocomial infections significantly more often than well-nourished patients, and they also have a longer hospital stay [20].

The median age of the patients included in our study was 110 months, which is higher than in previously published articles [7,17,21,22]. The most common underlying disease as the main indication for PEG insertion in our research is CNS disease, which is in correlation with other larger studies [18,21,22]. Although the most common indications for PEG placement are similar in most studies, the median age varies. More research is needed to analyze the relationship between age and efficacy and safety outcomes after PEG placement.

Many patients with severe neurological impairment have difficulty feeding [23,24]. Their energy consumption is usually too high, and their intake is deficient. PEG placement in these patients significantly improves malnutrition and does not cause major complications [25]. The median age of patients with cerebral palsy in our study was 12 years and 6 months, which is higher than published [18]. Feeding difficulties and undernutrition are high in patients with cerebral palsy. To prevent undernutrition, these children require adequate nutritional support and early enteral nutrition [26]. PEG significantly improves malnutrition in patients with cerebral palsy and does not cause major complications [18,25]. Earlier PEG placement should be considered in these patients to prevent malnutrition and improve patients’ quality of life. A study that enrolled 51 children showed that PEG is a safe technique, even in children weighing less than 10 kg, with complications that are comparable with older children [27].

A minimally invasive laparoscopic approach has been well described as safe and effective method in many different conditions in children [28,29,30,31,32]. Several studies showed that percutaneous gastrostomy placement has more common major complications than the laparoscopic method [33,34]. Our study included patients with percutaneous gastrostomy placement, but we recorded only one major complication (gastrocolic fistula) which required resection of the part of colon together with the fistula. This coud be the result of having a well-coordinated and experienced team at our hospital. The rate of minor complications is different among published studies. Some studies have similar incidence in minor complications to our studies [7,35], while others have more [19], or less, minor [22] complications. Since it is not clear yet what the risk factors for complications of development are, further studies are needed. The higher complication rate in our study could be associated with higher age of the patients, but it might be that, due to small sample size, we have not reached statistical significance.

Placing nasogastric tubes next to the bed in children can, due to incorrect placement, cause a number of complications. According to the United Kingdom National Health Service Improvement-issued safety alert titled “Nasogastric tube misplacement: continuing risk of death and severe harm”, the only safe confirmation of a well-placed tube is measurement of the pH [36,37]. Unfortunately, not all institutions are able to do that. Additionally, previous research recommend that nasogastric tube feeding should, due to discomfort and trauma for children, be limited to 3–6 weeks [3,35]. The median duration time for nasogastric feeding in our study was 11 months. This time should be shortened according to recommendations, and since the recent study has shown that PEG placement provides much greater benefits for children than nasogastric feeding [38].

The main limitation of our study is the lack of analysis of laboratory findings and its correlation with PEG complications. Other limitations are the relatively small sample size and retrospective character of the study. Anthropological data were recorded only at the time of PEG placement, therefore it was impossible to monitor the physical progress of the patients in this study. Additionally, there is a large age difference between the patients whose data were analyzed. However, this research provided new information about complications of procedures. Additional research is warranted to clearly determine the association between changes in laboratory findings, age and BMI with complications.

## 5. Conclusions

PEG is a safe and effective method for use in the pediatric population, with low incidence of complications. The most common indication for PEG placement was underlying CNS disease. At the time of PEG placement more than half of the patients had impaired BMI. Negative deviations of z-score body weight, body height, and body mass index could indicate the need for possible earlier placement of PEG.

## Figures and Tables

**Table 1:** Characteristic of the children who underwent PEG tube insertion.

Variable	Female Median (IQR)	Male Median (IQR)	P *
Body mass (kg)	22 (15, 29)	19 (11, 34)	
Body height (cm)	127 (100, 130)	119 (91, 139)	
BMI (kg/m^2^)	14.2 (13, 17)	15 (12, 19)	
z-score for body mass (kg)	−1.7 (−3.9, 0.52)	−0.30 (−4, 0.53)	0.583
z-score for body height (cm)	−1.5 (−2.8, −0.18)	−1 (−2, 0.96)	0.768
z-score for BMI (kg/m^2^)	−2 (−3, 0.28)	−0.67 (−4.2, 0.4)	0.894

* Mann–Whitney test, BMI–body mass index.

**Table 2:** Type and frequency of complications after PEG insertion.

Type of Complication	N (%) of Patients
Local infection	10 (55.5)
Granulation/scarring	4 (22.2)
Mechanical difficulties	3 (16.7)
Gastrocolic fistula	1 (5.6)

**Table 3:** Clavien–Dindo classification of surgical complications.

Grade, N (%)	Early Postoperative Complications	Late Postoperative Complications	Total
I	7 (41.2)	3 (17.6)	10 (58.8)
II	1 (5.9)	1 (5.9)	2 (11.8)
III a	1 (5.9)	3 (17.6)	4 (23.5)
III b	0 (0)	1 (5.9)	1 (5.9)
IV	0 (0)	0 (0)	0 (0)
V	0 (0)	0 (0)	0 (0)

**Table 4:** Analysis of age, BMI, and z-score for BMI, BM and BH in patients without and with complications related to PEG.

	No Complications, Median (IQR)	Complications, Median (IQR)	P *
Age (months)	101 (53, 171)	60 (42, 125)	0.101
BMI (kg/m^2^	14.2 (9.3, 22.8)	14.4 (11, 21)	0.935
z-score for BMI	−1.8 (−3.1, −0.8)	−1.7 (−3.9, 1.2)	0.730
z-score for BM	−1.5 (−4.7, −0.8)	−0.61 (−3.8, 0.44)	0.399
z-score for BH	−1.5 (−3.2, 0.14)	−1,7 (−2, 0.54)	0.99

* Mann–Whitney test, BMI–body mass index, BM–body mass, BH–body height, PEG–percutaneous gastrostomy.

## Data Availability

The data underlying this article will be shared on reasonable request to the corresponding author.
